# Designing a Nurse‐Led eHealth Cardiac Rehabilitation Program: Insights From Participant Experiences and Qualitative Feedback

**DOI:** 10.1111/phn.13437

**Published:** 2024-10-06

**Authors:** Jing Jing Su, Jenniffer Torralba Paguio, Weidi Wang, Ladislav Batalik

**Affiliations:** ^1^ School of Nursing Tung Wah College Hong Kong China; ^2^ College of Nursing The University of the Philippines Manilla Philippines; ^3^ Department of Nursing Tongji Hospital of Tongji Medical College Huazhong University of Science and Technology Wuhan China; ^4^ Department of Rehabilitation, University Hospital Brno and Faculty of Medicine Masaryk University Brno Czech Republic; ^5^ Department of Physiotherapy and Rehabilitation, Faculty of Medicine Masaryk University Brno Czech Republic; ^6^ Department of Public Health, Faculty of Medicine Masaryk University Brno Czech Republic

**Keywords:** behavioral change, cardiac rehabilitation, coronary heart disease, eHealth, patient‐centered care

## Abstract

**Background:**

This study examines the perspectives of individuals with coronary heart disease (CHD) on a nurse‐led eHealth cardiac rehabilitation (NeCR) program, which included a website, tele‐monitoring device, and social media chatroom.

**Methods:**

Using a descriptive qualitative approach, semi‐structured interviews were conducted with 18 participants to capture their feedback and experiences with the NeCR program.

**Results:**

Participants found the NeCR program valuable in addressing gaps in cardiac rehabilitation services in China, empowering them to make behavioral changes and enhancing their social motivation. However, they also highlighted the need for a more user‐friendly website, better symptom management during exercise, and stronger privacy protections in the peer networking chatroom. The study concludes that the NeCR program is feasible in providing accessible rehabilitative services at home post‐discharge. Recommendations include improving the self‐monitoring platform for ease of use, incorporating immediate symptom management guidance during exercise, and ensuring a secure environment for online peer support.

**Conclusions:**

These findings offer crucial insights for developing patient‐centered eHealth cardiac rehabilitation services, emphasizing the importance of user‐friendly design, effective symptom management features, and privacy protection in promoting participant engagement with e‐platforms.

**Trial Registration:**

ChiCTR1800020411 (http://www.chictr.org.cn/showprojen.aspx?proj=33906)

## 1 Introduction

The British Heart Foundation recently released a report estimating that more than 250 million people worldwide are living with coronary heart disease (CHD) (British Heart Foundation 2024). CHD results in detrimental outcomes, causing individuals to frequently experience symptoms, stress, subsequent cardiac events, and a decreased quality of life (QoL). The resource constraints of the healthcare system, competing priorities of patients, and cumulative exposure to modifiable CHD risk factors (e.g., stress and sedentary lifestyle) make cardiovascular care challenging. Involving patients in their care and designing interventions that incorporate their beliefs, preferences, and customs, along with recommended clinical guidelines, is essential to address these challenges effectively (Goldfarb et al. 2024).

Cardiac rehabilitation (CR) is a supervised therapy that encompasses patient education, exercise, and psychosocial support to slow disease progression and prevent cardiac recurrences in patients with CHD (Thomas et al. 2019). Evidence indicates that CR programs effectively enhance physical activity levels, control cardiovascular risk factors, improve QoL, and reduce cardiovascular mortality and re‐hospitalizations in patients with CHD (American Association of Cardiovascular and Pulmonary Rehabilitation 2021; Campos et al. 2022; Thomas et al. 2019). Despite the clinical benefits of CR, accessibility and adherence to conventional CR remain poor. Globally, half of the countries do not offer CR services; and only 15%–35% of eligible patients participate in CR interventions (Fang et al. 2017; Turk‐Adawi et al. 2019). The positive association between CR availability and utilization underscores the need for integrating alternative models to enhance its use (Fang et al. 2017; Turk‐Adawi et al. 2019).

Offering CR via information and communication technology, known as eHealth CR, has been advocated as an alternative method to promote home‐based CR, which relies on remote supervision and tele‐coaching rather than traditional center‐based programs (Stefanakis et al. 2022; Su, Yu, and Paguio 2020). The components of CR can be delivered through various technologies, such as mobile applications, websites, monitoring sensors, instant messages, and social media (Su, Yu, and Paguio 2020). eHealth CR enables patients to access health modules, upload health data with automated feedback, acquire disease management skills, and seek support from peers and healthcare professionals without the limitations of time and location (Schwamm et al. 2017). This method facilitates a more patient‐centered and autonomous approach by allowing personalized action plans, self‐care monitoring, and progress visualization on individual web pages to cater to specific needs (Brewer et al. 2017; Widmer et al. 2017). Moreover, the extensive use of behavior changes techniques—such as goal setting, tele‐monitoring, and motivational feedback—has been recognized as beneficial features for effective eHealth CR interventions (Su, Yu, and Paguio 2020).

However, previous eHealth CR studies have reported significant user disengagement or non‐usage attrition, where participants participated in the study but did not actively use the eHealth platforms (Dale et al. 2015; Engelen et al. 2020). This disengagement can undermine the validity of cause‐effect conclusions related to eHealth CR interventions. Patient engagement, defined as the active use of e‐platforms to optimize health outcomes, is central to the success of eHealth CR interventions (Su, Yu, and Paguio 2020; Thomas et al. 2019). User disengagement may stem from a lack of patient involvement in the design and feedback processes to improve the user‐friendliness and acceptability of these platforms. Exploring user feedback during the development of eHealth interventions is crucial to understanding patients’ information needs and refining content delivered by complicated technology methods (e.g., telemonitoring and website). For instance, one study incorporated patient feedback both before and after establishing a CR website to ensure the educational information was understandable and user‐friendly (Pfaeffli et al. 2012). Another study gathered feedback from patients with CHD who used a 6‐week online CR information self‐help manual, which enhanced the accessibility, readability, and user‐friendliness of the online material (Deighan et al. 2017). Additionally, a cardiac tele‐rehabilitation study interviewed patients about their experience using a pedometer to motivate physical activity at the end of a 12‐week intervention (Thorup et al. 2016). However, collecting feedback solely on the use of pedometers is insufficient, as emerging studies suggest that a combination of wearable devices, graphic visualizations, educational content, and teleconsultation provides a more comprehensive CR experience (Su, Yu, and Paguio 2020).

The aforementioned studies are crucial for informing the development and refinement of e‐platforms; however, they have limitations due to the lack of guiding theory and the comprehensive integration of behavior change techniques (e.g., data uploading, tele‐monitoring, motivational feedback, and social networking). Additionally, there are contradictions regarding the adoption of synchronized social media platforms for facilitating direct individual/group peer and professional interactions from the perspectives of healthcare professionals and researchers (Shen et al. 2019). The primary concerns revolve around confidentiality and the potential for these platforms to rapidly disseminate negative emotions and doubts. Furthermore, the common challenge of non‐usage attrition reported in previous eHealth CR programs (Dale et al. 2015; Engelen et al. 2020) highlights the importance of gathering patient feedback and experiences from the outset. To address these issues, a new nurse‐led eHealth CR intervention, titled “NeCR,” has been designed. This intervention includes a brief in‐person preparation session followed by an extensive e‐platform use session. The e‐platform comprises a comprehensive CR website, a pedometer for daily step monitoring, and a widely accepted social media app (WeChat) for peer interaction and expert consultation, tailored for Chinese patients with CHD (Su and Yu 2019). This study aims to explore the perspectives of patients with CHD on the newly developed NeCR program and to refine it before its implementation in a full‐scale randomized controlled trial.

## 2 Materials and Methods

### 2.1 Design

A qualitative descriptive design was employed for this study. Semi‐structured individual in‐depth interviews were conducted to explore patients' perspectives on the NeCR intervention. This method permits in‐depth exploration of individual participants' experiences and their constructed perceptions of the NeCR intervention (Holloway and Galvin 2016). Individual interviews were held 2 weeks after the participants began using the NeCR intervention, utilizing video calls to capture their initial experiences and feedback. The 2‐week timeframe was intentionally chosen to allow participants ample opportunity to engage with all components of the intervention, such as weekly/daily data uploads to the CR website, nurse consultations for goal attainment, and participation in a chatroom for progress sharing.

### 2.2 Participants

This study was conducted from January to March 2019 in a medical cardiac unit of a first‐rank tertiary hospital in Wuhan, China, where CHD patients are treated with percutaneous coronary intervention (PCI) and/or medication. The inclusion criteria for the study were as follows: adults with a diagnosis of CHD, no prescribed exercise restrictions, access to the Internet at home, and a minimum primary education level. Only participants who had completed an individual assessment for goal setting and logged into the CR website were interviewed. Exclusion criteria included patients diagnosed with a life‐limiting condition, those with acute mental illness, individuals with relative or absolute contraindications to exercise training, and patients enrolled in another secondary prevention program. A total of 167 cardiac patients were initially screened for the study. Out of these, 31 patients met the eligibility criteria and were subsequently invited to participate. Ultimately, 18 patients consented to participate and were interviewed.

### 2.3 Ethics Considerations

The study was carried out in compliance with the Helsinki Declaration of Ethical Principles for Medical Research, in which human subjects participate, and was approved by The Joint Chinese University of Hong Kong—New Territories East Cluster Clinical Research Ethics Committee (2018.469). Permission was also obtained from the study hospital. All participants provided written informed consent before participation. Participants were allowed to continue using the telemonitoring and CR website after study completion.

### 2.4 Intervention: The Nurse‐Led eHealth Cardiac Rehabilitation

Participants began the intervention approximately 1 week after their acute event, during the last 2 days of their hospitalization. The NeCR intervention was comprehensively detailed in a published protocol (Su and Yu 2019). Guided by social cognitive theory, the NeCR began with a brief in‐person assessment conducted by a cardiac nurse. This assessment included counseling on physical activity, fitness habits, nutritional and psychosocial management, smoking cessation, and social factors affecting disease management. The nurse evaluated each participant's exercise capacity using a 6‐min walk test, monitored by remote electrocardiography observed by another nurse. Based on these assessments, the nurse calculated the metabolic equivalent (MET) level and introduced guideline‐based recommendations to assist patients in setting goals and action plans tailored to their personal risks and preferences. These goals were then uploaded to the patients' personal websites. The goal setting for exercise was designed to be progressive, with the aim of gradually increasing the exercise dosage to achieve at least 150 min of moderate exercise per week. The action planning was more specific, such as prescribing brisk walking for 30 min every day after dinner. Similar collaborative processes were used for goal setting and action planning regarding diet, stress management, and smoking cessation. For instance, one participant with a high‐stress score of 7 set a goal to reduce it below 4 and planned to practice progressive muscle relaxation three times a week. Furthermore, the nurse organized group sessions to familiarize participants with the website and instruct them on using a pedometer. Participants were then asked to connect to a social media chatroom. Each participant was provided with a goal‐directed action plan and a user manual detailing the use of the website, telecare platform, and pedometer.

The CR elements were delivered through a password‐protected web application and a concurrent chatroom to engage participants and promote behavioral modification. The content was based on updated international guidelines (American Association of Cardiovascular and Pulmonary Rehabilitation 2021) and reviewed and validated by CR professionals, including nurses, cardiologists, physiotherapists, and dietitians to ensure its accuracy and appropriateness for CHD patients. Participants were encouraged to upload data on their physical activity, diet, stress levels, and smoking behaviors to the self‐monitoring platform on a weekly basis. They could log weekly exercise time, duration, and frequency, indicate their stress level on a scale from 0 to 9, check their dietary score, and submit their smoking status. Additionally, participants were encouraged to wear a pedometer daily and upload their step data to the website. A traffic light system, featuring encouraging messages and a trending graph on overall goal attainment, was used to promote positive behavioral changes. The health modules presented comprehensive CR content for learning and skills acquisition, including symptom observation (e.g., chest pain/heaviness, advice on post‐PCI activities), physical exercise, nutrition, psychosocial management, tobacco smoking cessation, education about CHD, and risk factor control (e.g., hypertension, dyslipidemia, and diabetes). The web content adhered to the Health Literacy Online guidelines to enhance understandability (Office of Disease Prevention and Health Promotion 2016). The nurse collected self‐monitoring health data from the website and anonymously shared each participant's weekly goal‐attainment progress via the chatroom. Participants were encouraged to interact with peers and nurses for experience sharing and consultation. Queries and conversations were promptly replied to and moderated by the nurse, usually within 1 h, from 8 am to 8 pm during the intervention period.

### 2.5 Data Collection

The lead researcher (J.J.S.), a female PhD candidate in nursing with extensive qualitative interviewing experience, conducted individual interviews with 18 participants via video calls following a 2‐week participation in the NeCR program. The researcher had no prior relationship with the patients and conducted the interviews in Mandarin, adhering to a guide designed to explore the participants' perspectives on the NeCR program (see Table 1). The use of video calls facilitated discussions in the natural settings of the participants (e.g., their homes), aligning with the notion that the context in which a phenomenon occurs is integral to the phenomenon itself (Saarijärvi and Bratt 2021). Each interview was audio‐recorded and lasted approximately 30–60 min. To ensure participant comfort, interviews were scheduled at the patients' preferred times, with the researcher observing for signs of fatigue and providing emotional support as needed. Field notes were documented during and immediately after the interviews, capturing observations of the setting and non‐verbal expressions. Data saturation was achieved by the 15th interview and was confirmed through three additional consultations.

**TABLE 1:** Interview guide.

How do you evaluate the NeCR program? (The website, the pedometer, and the WeChat component). Please share your thoughts about the face‐to‐face session. [Probes: thoughts on individual assessment and counseling, the orientation session]. Please share your thoughts about the NeCR website. [Probes: thoughts on self‐monitoring, diet, psychosocial management, exercise, and smoking cessation web‐platform]. How do you comment on the ease of use of the NeCR? (The website, the pedometer, and the WeChat). Why do you think it is easy or difficult to use? What do you think of the presentation of the website? (The content, format, language, and layout). Why do you find it positive or negative? Please share your thoughts about your interactions and communication with the nurse. How do you feel about it? Please share your thoughts about your interactions and communication with your peers. How do you feel about it? Please explain how this NeCR program influences the way you manage coronary heart disease. Please explain how this NeCR program influences your lifestyle behaviors.

### 2.6 Data Analysis

Thematic analysis was used to analyze data. The first author reviewed the recordings repeatedly to immerse in the data. Verbatim transcriptions of the recordings were reviewed and checked for accuracy against the original recordings. The transcripts were translated into English by the first author and cross‐checked by another researcher who is bilingual. Conceptually related codes were grouped into sort categories. The researchers then searched for structures and patterns to connect the categories to generate themes (Shenton 2004). A discussion among authors (J.J.S. and J.P.) led to a consensus on the generated codes, categories, and themes.

### 2.7 Trustworthiness

A robust approach to data collection and analysis was employed to ensure credibility. All interviews were audio‐recorded and securely stored. Only participants who had used the NeCR were interviewed, as they were considered to possess firsthand knowledge about the intervention, thereby ensuring credibility. The interviews focused on the use and experience of the NeCR intervention, enabling the responses to be recontextualized and potentially transferred to different eHealth CR delivery settings. The researcher listened to the recordings repeatedly to verify the accuracy of the transcripts. A neutral and non‐judgmental tone was maintained throughout the interviews to avoid influencing participants or eliciting more favorable responses. Transferability was confirmed through detailed descriptions of participants' experiences and perceptions after using NeCR. In line with Shenton A.’s study (Shenton 2004), constructs and an audit trail were utilized to compare personal biases and critically reflect on practices. Recordings and field notes were referenced to assess the credibility and trustworthiness of the findings.

## 3 Results

Eighteen patients hospitalized for CHD were included. The demographic and clinical characteristics of the participants are presented in Table 2. The participants had a mean age of 55.8 years (SD = 6.2), with the majority being male (77.8%) and reporting daily Internet use (88.9%). Most participants underwent PCI (77.8%), and a significant portion had comorbid conditions such as hypertension (33.3%), diabetes (38.9%), and dyslipidemia (55.6%). Overall, participants reported feeling supported and guided by the NeCR program in terms of behavior change and disease management. They affirmed the usability and acceptability of NeCR in addressing the gaps in limited CR services in China, empowering active behavioral change, and enhancing social motivation for such changes. However, participants also highlighted the need for strengthened content on symptom management during exercise, improvements to make the NeCR e‐platform easier to interact with, and concerns regarding the potential invasion of privacy.

**TABLE 2:** Demographic and clinical characteristics of the participants (*N*  = 18).

Patient characteristics	*N* (%)/M (SD)
Age	55.8 (6.2)^a^
Male	14 (77.8%)
Years of education	9.4 (2.7)^a^
Married	16 (88.9%)
Numbers of families living in the same household	3.3 (1.6)^a^
Employment status	
Labor worker	6 (33.3%)
Retired	6 (33.3%)
Office worker	6 (33.3%)
Drinking alcohol	6 (33.3%)
Current tobacco use	6 (33.3%)
Daily Internet use	16 (88.9%)
Functionalities	
Social‐networking	17 (94.4%)
News and information reading	10 (55.6%)
Videos	6 (33.3%)
Computer use	7 (38.9%)
Phone use	15 (83.3%)
Treatment	
Percutaneous coronary intervention	14 (77.8%)
Medication	4 (22.2%)
Diseased vessels	
Single	7 (38.9%)
Multiple	11 (61.1%)
Hypertension	6 (33.3%)
Type 2 diabetes	7 (38.9%)
Fasting blood glucose mmol/L	8.1 (3.9)^a^
Dyslipidemia	10 (55.6%)
Body mass index (kg/m^2^)	
Overweight 25–30	8 (44.4%)
Obesity >30	2 (11.1%)

^a^ Mean (SD).

During the 2 weeks of intervention usage, 88.8% of participants uploaded health data on exercise and diet on the website, while only 22.2% reported stress scores. The pedometer‐measured daily steps increased by an average of 3078.8 steps (SD = 2282.6) from baseline to 2 weeks among 16 participants. All participants joined the teleconsultation chatroom except two who refused for confidentiality concerns.

**FIGURE 1:**
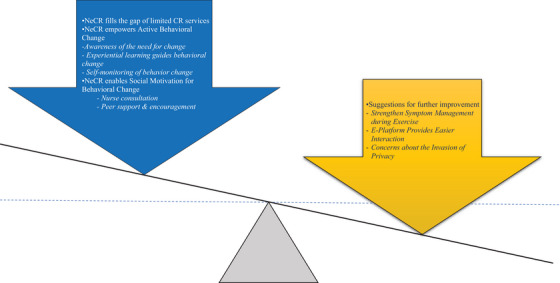
Summation of patients’ experiences and feedback. [Colour figure can be viewed at wileyonlinelibrary.com]

Three themes emerged characterizing the patients’ experiences of and feedback for the NeCR intervention, which can be broadly dichotomized into facilitators and barriers. The facilitators empowered patients to engage in the intervention and initiate lifestyle behavior change, including (1) NeCR fills the gap of limited CR services in China and (2) NeCR empowers active behavioral change. In contrast, the barriers identified for further improvement include the need for strengthened symptom management during exercise, enhancements to make the e‐platform easier to interact with, and concerns regarding privacy invasion. A summation of patients’ experiences and feedback is illustrated in Figure 1.

### 3.1 NeCR Fills the Gap of Limited CR Services in China

All participants recognized that the NeCR program supplements the limited healthcare access to institutions and healthcare professionals with CR knowledge and expertise to facilitate disease management post‐discharge. They expressed that, after discharge, they were supposed to reassume their previous social and familial roles on their own without rehabilitative support. Participants considered the NeCR program as a continuity of professional care facilitating their transition from hospitalization. Some expressed limited access to programs like the NeCR and were “privileged to receive such service from a research hospital.” They were pleased that the NeCR program platform allowed them to save on travel expenses and care services in managing CHD. The participants shared the following:

“I came from a remote area. Health workers in my area do not know about stent (care). They refuse to give me care and ask me to visit the hospital once they heard I had received it (PCI) from a big hospital. I need this online rehab thing. It saves my cost to seek consultation.” (P2)

“I was in good health. I have never been sick or hospitalized. (I) Never thought this was something serious until hospitalization. This program helps me to take care of myself. Only a hospital good as yours can provide such a program.” (P7)

“I like that it is free. I guess the web is costly (but) there is no advertisement. (The program is) Not for money. It is only for the patients’ wellbeing.” (P11)

### 3.2 NeCR Empowers Active Behavioral Change

The participants highlighted how eHealth CR can promote behavioral change. They shared that the NeCR increases awareness of the need for change, provides experiential learning to guide behavioral change, and enables self‐monitoring.

#### 3.2.1 Awareness of the Need for Change

Most participants described having limited knowledge of the risk factors of CHD before entering the NeCR program. They reported that the NeCR's individual assessment and counseling increased their awareness of personal risk factors and lifestyle problems, which motivated them to engage in behavioral change. They described their newfound understanding:

“I drink a lot, social drinking for business, hospitalized (for heart attack) after heavy drunk.” (P15)

Participants also described that goal‐setting and action‐planning reinforced their awareness of the need for behavioral change and the process.

“I became conscious about my goals!” (P6)

#### 3.2.2 Experiential Learning Guides Behavioral Change

Some participants shared that they sought health information from print, television, and online media and encountered limited professional guidance on CHD management. Accessing credible CR information through a readily accessible website was perceived as motivation for NeCR participation. Participants shared the importance of learning how to prevent a second cardiac event from reliable NeCR websites, unlike those offered by laypersons, advertisers, and third‐party companies. They found various core components covered on the NeCR website to be useful health resources and decision‐making support, such as early mobilization after PCI, dietary recommendations for CHD and diabetes management, and managing alcohol consumption. The participants described:

“This is the second time I have received a stent. Scary! I used to read online about it. The terminology and rumor were useless. I think the NeCR website has a good focus on actions. What I can do and how to do it. It's more straightforward.” (P2)

“The website diagram said alcohol drinking is bad and may interact with medications. I quit.” (P7)

#### 3.2.3 Self‐Monitoring of Behavioral Change

All participants unanimously shared the motivation they derived from telemonitoring and the sense of achievement they felt when meeting their daily steps goals. They also noted that they felt better during and after physical activity, such as having increased energy and decreased symptoms, which motivates behavioral change.

“I felt my heart rate had increased when I walked. I could tolerate it. Then I increased my walking time. I have just started jogging ten minutes a day.” (P1)

### 3.3 NeCR Enables Social Motivation for Behavioral Change

#### 3.3.1 Nurse Consultation Guides Behavioral Change

Participants felt reassured by having immediate access to the nurse when they encountered challenges related to behavioral change and symptom management. They appreciated the direct and instantaneous communication with the cardiac nurse through various channels, such as voice messages, phone calls, and sending photos (e.g., medical records, medication, and side effects on the skin) via WeChat. All participants expressed gratitude for the nurses’ assistance when they were unsure about their lifestyle and disease management. Their engagement with the nurse helped them continue with their predetermined goals and plans. Two participants shared their experiences:

“I was upset about chest heaviness. I called (nurse). I learned how to monitor my pulse and symptoms.” (P5)

“I started to exercise the first week after discharge. I sweat a lot after walking for about 20 minutes. I talked to the nurse. I think such consultation is essential, especially when we have just been discharged from the hospital.” (P2)

#### 3.3.2 Peer Support Encourages Behavioral Change

Participants who joined the WeChat group reported reading all the progress updates and health dialogue on the group chat. They noted that being hospitalized and discharged at a similar time with a diagnosis of CHD made their concerns and experiences particularly relevant to one another. They expressed interest in others' progress and experiences of behavioral change, as well as the health dialogues between nurses and peers. One participant shared the following:

“I read all the chatroom information. I wanted to see how others recovered, how the nurse responded to others' queries, and how others progressed in goal attainment.” (P1)

Participants described that they would communicate with the group to see if others had similar experiences when encountering particular symptoms.

“One day, my nose suddenly bled. Nervous! I asked in the chatroom. Other patients shared their side effects of anti‐coagulant treatment. It reassured me.” (P7)

### 3.4 Suggestions for Further Improvement

Apart from the above‐mentioned advantages, participants also provided suggestions to improve the intervention, focusing on enhancing symptom management during exercise, strategies to improve website interaction, and concerns about privacy.

#### 3.4.1 Strengthen Symptom Management During Exercise

A few participants perceived the recurrence or persistence of cardiac symptoms as a threat to performing activities, such as palpitations and sweating during physical activity, and sudden bruising or bleeding. Participants who underwent PCI consistently expressed their need for more guidance in understanding and managing symptoms after discharge to perform exercise. Some participants shared these statements:

“I sweat a lot after 2000 steps of slow walking. I did not know if it's my heart having another infarction or it's the side effect of my stent.” (P2)

#### 3.4.2 E‐Platform Provides Easier Interaction

Understanding the NeCR program's potential benefits, some older participants highlighted the ease of interacting with the website, which allowed them to use self‐care monitoring protocols and upload their results for feedback. Two participants stated:

“I wanted to assess my stress level once. When I moved the bar, I meant to (go to) the middle, but I ended up on the right side. Red light popped up (indicating) extremely stressful. Error! It's better if it is easier with a larger touch space.” (P17)

“I have to type in several digits to fill in daily steps. It's easy to touch the wrong number.” (P13)

Some participants suggested changes in navigating the website, such as having more visible navigation buttons and increasing pathways or links, so that users could browse information more thoroughly and identify needed information quickly. One participant desired easy navigation for learning:

“I am getting old. I may need help finding the navigation button. Consider making it more obvious?” (P12).

Participants appreciated the ability to track their progress by visualizing their data history.

“I liked the graph showing my daily steps; it reflected my progress.” (P14)

#### 3.4.3 Concerns About the Invasion of Privacy

Two patients were reluctant to participate in the face‐to‐face group sessions and group chatroom because of privacy and security concerns and peer distrust. One participant stated:

“I don't want others to know about my illness online. If other patients know me and my contact methods on WeChat, they may spread information.” (P14)

Some participants in the WeChat group expressed initial hesitation about extending their online interaction to direct discussion. They also expressed the need to see a clear information‐sharing scope.

“I was conservative at the beginning. After reading the dialogue for days, I can see a clear health focus.” (P1)

Since the WeChat platform was open for both participants and the nurse, some participants expressed concern over the credibility of the information provided by their peers. They mentioned that their peers might provide unreliable and misleading information, which may have detrimental consequences or result in unnecessary worries. They shared:

“If I do not know something, how come other patients know it? Besides, it's good to have an expert address the concerns quickly before everyone in the group gets misinformed.” (P2)

## 4 Discussion

The patients shared their experiences and feedback after the initial use of a comprehensive NeCR program. They appreciated the NeCR for providing accessible and affordable CR services with minimal geographical barriers. This acceptance can be partially attributed to the perceived symptom burden and the lack of phase II CR services in China following a cardiac event. Participants highly valued the individual assessments that identified personal risk factors and helped form individual goals and action plans, as well as the consultation support provided in realizing these action plans. They suggested specific modifications to the e‐platform for easier navigation, operation, and comprehension, enabling them to use the website whenever needed. Since the CR website is guideline‐based and professionally validated, participants did not challenge the content; rather, they expressed a need for more information on post‐PCI management. Reluctance to participate in online peer support chatrooms due to privacy concerns highlights the necessity of establishing a secure peer network forum. This forum should have a clear health purpose and defined information‐sharing boundaries, moderated by professionals

Consistent with the literature, participants valued the home‐based nature of the NeCR intervention, which offers a solution for providing accessible and affordable CR services in China with minimal logistical barriers (Thomas et al. 2019). The intervention is timely, as patients post‐PCI experience various symptoms such as functional decline, pain, depression, shortness of breath, and tiredness (Barker et al. 2018). The program design should include substantial symptom management information related to monitoring abnormal symptoms associated with CHD and PCI. Participants also requested additional content on monitoring and interpreting symptoms related to exercise. Thus, guidance on monitoring, interpreting, and decision‐making regarding physical symptoms during exercise should be improved. Internationally, eHealth CR has significantly improved CR uptake and has been incorporated into the healthcare systems of several countries, including Australia, Canada, and the United Kingdom (British Association for Cardiovascular Prevention and Rehabilitation 2018; Pecci and Ajmal 2021). As e‐platforms have been increasingly used in healthcare provision during the COVID‐19 pandemic, translating such a care model into routine practice is timely and necessary (Babu et al. 2020).

To make such a model attractive, participants desired an e‐platform with easier interaction which aligns with existing studies emphasizing human–computer interaction to enhance user experiences and adherence (Søgaard Neilsen and Wilson 2019; Su et al. 2023). Participants recommended having more obvious navigation buttons or additional web links, supporting previous studies where users expressed the need for more explicit and clear instructions (e.g., access to internal web links) to facilitate navigation and enhance usability (Olmsted‐Hawala et al. 2018). Participants also highlighted the need for more effortless operation when entering health data on a smartphone, considering the small touch‐screen space, which is critical for older adults with reduced vision and psychomotor function (Olmsted‐Hawala et al. 2018). Feedback from the participants led to modifications of the website prototype, such as the choice to select instead of keying in data, adjusting font size, and enlarging selection buttons. This feedback was provided to the technician to amend the prototype, making it more feasible, persuasive, and relevant to the designated users (Findeis and Patyk 2020). These findings underscore the importance of inviting participants' feedback in designing eHealth interventions. Modifying the readability level of online health information is inadequate given the current use of multiple media formats (e.g., video, audio, and graphics) and the wide age range of CHD patients. Beyond generic interface design tips on font size or navigation buttons, the underlying emphasis is that each intervention component should be clear and explicit to guide patients toward health benefits without overwhelming them with presentation skills.

Findings from this study supported the use of a hybrid approach that includes individual assessment and goal setting rather than starting directly with the e‐platform. A fundamental requirement to motivate eHealth CR usage is that the program should address a problem that the patient has identified and can help resolve (van Velsen et al. 2019). This approach provides eHealth engagement with clear goals and orientation, which determines the strength of its usage (Chiang et al. 2018). The individual assessment and goal setting assist patients in identifying self‐care deficits and recognizing the potential benefits of program participation. The NeCR website provides comprehensive and actionable guidance, functioning as a resource to guide behavior modification and address those deficits.

The findings affirm the role of social support in facilitating behavioral change through nurse consultation and nurse‐moderated peer interaction. Nurses are highly competent primary care providers who can work with patients of different capacities and preferences (Kingod et al. 2017) and are in critical positions to identify challenges and partner with patients remotely. The results support the presence of a nurse moderator to accompany patients through the rehabilitation journey, addressing concerns raised in the literature about peer chatrooms potentially being filled with concerns, negative emotions, and uncertainties (Houle et al. 2020).

Patients' feedback on privacy issues echoed the concerns of professionals and academics, given the widely reported illicit collection of detailed personal data and technology‐based information leakage in healthcare (Antypas and Wangberg 2014). Previous eHealth CR studies have made attempts to make peer‐networking optional, such as allowing peers to post progress and experiences (Southard, Southard, and Nuckolls 2003) and sharing email addresses (Su et al. 2020). Findings from this study suggest precautions for integrating more direct peer networking through social media. Providing a structured peer support platform with a clear illustration of its purpose, frequency, communication strategies, and anonymity may be crucial. Establishing a secure peer networking environment where participants agree to protect each other's privacy and understand the boundaries of information sharing is critical for effective peer interaction. Considering the increasing focus on privacy worldwide, exemplified by the General Data Protection Regulation (GDPR) in Europe (European Council of the European Union 2022), using social media for peer networking should be optional.

### 4.1 Implications

Several strategies can be used to secure peer interaction and promote greater participation in the e‐platform. These include ensuring that no personal information is exchanged during peer interactions (both face‐to‐face and web‐based), focusing discussion content on behavioral goal attainment, and restricting membership to only the nurse and assigned members. The telecare platform also functions as a consultation platform where nurses moderate discussions and respond to patients. eHealth CR interventions should provide an individual assessment to identify personal risk factors and form individual goals and action plans at the commencement of the program. Inviting participants' feedback could be a useful strategy to modify the e‐platform for easier navigation, operation, and comprehension. Additionally, guidance on post‐PCI management is essential for participants to begin using the intervention and ensure their engagement in physical activity.

### 4.2 Limitations

Sample recruitment from a single healthcare institution in China, in response to a specifically designed NeCR, may limit the transferability and richness of the findings. The results may not be applicable to other healthcare settings using different eHealth CR intervention designs. This study included and interviewed participants who used the NeCR exclusively, who might be relatively positive toward the program. Future studies may explore the views of eHealth CR among underutilized populations, such as women and older adults with CHD, to improve service accessibility. Despite these limitations, the results provide valuable insights into the further development and provision of eHealth CR interventions and large‐scale intervention testing. The short period of NeCR usage may limit the richness of data that could emerge from longer‐term use of the platform

## 5 Conclusions

Participants perceived the NeCR program as a solution to the currently limited rehabilitation services in China, facilitating home‐based self‐care and disease management following acute cardiac events. They emphasized the importance of comprehensive empowerment strategies for activating self‐care behavior and expressed a desire for instant and specific symptom management guidance during exercise. Furthermore, a secure and structured online environment is necessary to encourage participation and engagement in peer networking platforms.

## Author Contributions

All authors contributed to the conceptualization, data analysis, manuscript writing, and revision. Data collection was conducted by J.J.S. All authors contributed to editorial changes in the manuscript. All authors read and approved the final manuscript.

## Ethics Statement

The Joint Chinese University of Hong Kong—New Territories East Cluster Clinical Research Ethics Committee (2018.469) issued this study's ethical approval. All participants provided written informed consent before participation.

## Consent

The author has nothing to report.

## Conflicts of Interest

The authors declare no conflicts of interest.

## Data Availability

Data are available upon reasonable request. The data set used are available from the corresponding author upon reasonable request.
